# Development of a Blocking ELISA for Detection of Serum Neutralizing Antibodies Against Duck Adenovirus Type 3

**DOI:** 10.3390/microorganisms13112607

**Published:** 2025-11-16

**Authors:** Mei Tang, Xiaona Shi, Yifei Xiong, Chunxiu Yuan, Qinmin Zhu, Minfan Huang, Bangfeng Xu, Qinfang Liu, Xue Pan, Zhifei Zhang, Qiaoyang Teng, Minghao Yan, Dawei Yan, Zejun Li

**Affiliations:** 1 Department of Avian Infectious Diseases, Shanghai Veterinary Research Institute, Chinese Academy of Agricultural Sciences, Shanghai 200241, Chinashixiaonare@163.com (X.S.); yuanchx@shvri.ac.cn (C.Y.);; 2 College of Animal Sciences, Fujian Agriculture and Forestry University, Fuzhou 350002, China; 3 College of Veterinary Medicine, South China Agricultural University, Guangzhou 510642, China

**Keywords:** duck adenovirus type 3 (DAdV-3), monoclonal antibody, blocking ELISA, detection

## Abstract

In 2014, Duck Adenovirus type 3 (DAdV-3) emerged in Muscovy ducks and has since spread rapidly across China, causing significant economic losses to the duck industry. Given this situation, the development of reliable diagnostic tools is crucial for effective disease control. In this study, a neutralizing monoclonal antibody (mAb) 2F12 specific to DAdV-3 was generated, which showed a blocking rate of over 70% and a neutralization titer of up to 1:794. A blocking enzyme-linked immunosorbent assay (b-ELISA) was further developed based on mAb 2F12 to efficiently detect neutralizing antibodies against DAdV-3. The cut-off values of percent inhibition (PI) were set based on testing 84 negative duck serum samples, with a value below 16.79% (mean ($\bar{X}$) + 2 standard deviations (SD)) for negative sera and over 21.62% ($\bar{X}$ + 3SD) for positive sera. The b-ELISA exhibited a high specificity, reacting exclusively with DAdV-3 positive serum and showing no cross-reactivity with other representative positive sera tested. Additionally, the b-ELISA showed significantly higher sensitivity than the serum neutralization test (SNT), detecting antibodies 16-fold greater than the endpoint dilution of the SNT. The established b-ELISA, validated with 90 field serum samples from six duck farms, was well-suited for clinical detection of DAdV-3 antibodies and for monitoring post-vaccination antibody levels, representing a significant advancement in DAdV-3 detection and prevention.

## 1. Introduction

The family *Adenoviridae* contains a diverse group of viruses capable of infecting multiple animal species and humans [1]. As per the taxonomy issued by the International Committee on Taxonomy of Viruses (ICTV), published in 2024, the family *Adenoviridae* is currently divided into 6 distinct genera (*Aviadenovirus*, *Mastadenovirus*, *Atadenovirus*, *Siadenovirus*, *Ichtadenovirus*, and *Testadenovirus*) (https://talk.ictvonline.org/). Duck Adenoviruses (DAdVs) comprises DAdV-A (also termed DAdV-1) in the genus *Atadenovirus* and DAdV-B (comprising DAdV-2 through DAdV-6, the latter (DAdV-6) currently proposed rather than formally approved) in the genus *Aviadenovirus* [2,3,4]. In 2014, DAdV-3 was identified in Muscovy duck and widely spread across China [5,6]. And the infected ducks showed severe hepatic and renal damage, characterized by swollen, yellowing and hemorrhages of the liver, as well as slight pericardial effusion and hemorrhages in the kidneys [7]. The high incidence rate (40–55%) and mortality rate (35–43%) of DAdV-3 infections pose a substantial threat to duck industry [8,9].

**Figure 1:**
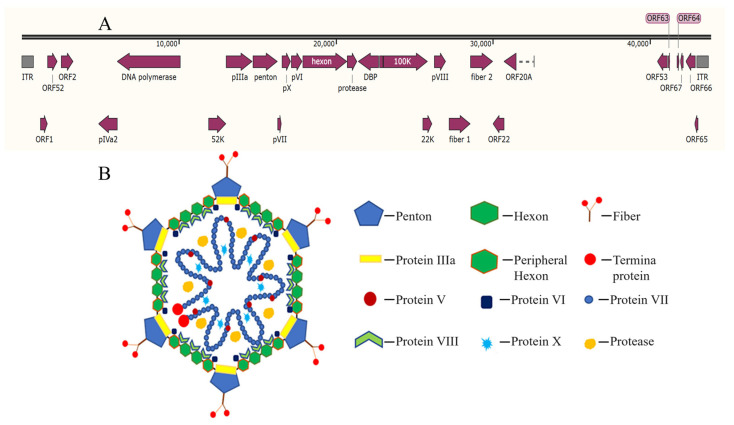
Genomic (**A**) and proteins (**B**) [15] structure of fowl adenovirus.

DAdV-3 is a non-enveloped, icosahedral DNA virus with a double-stranded, linear genome of approximately 44 kb in length (Figure 1A) [10]. It encodes three main surface structural proteins, including Hexon, Penton base and Fiber (Fiber 1 and Fiber 2) (Figure 1B) [11]. Hexon, as the main component of the viral nucleocapsid, constitutes approximately 60% of the total mass of viral particles and contains the specific antigenic determinants that induce neutralizing antibodies [12]. Penton is located at the top of the icosahedral capsid of the virus particle, connecting Hexon and Fiber, and plays an important role in stabilizing the virus capsid. During avian adenovirus infection, the Fiber 1 protein is the first to contact with host cells and mediates the interaction between the virus and cell receptors, the Fiber 2 protein governs virulence, drives protective immunity, and serves as a definitive diagnostic marker, making it central to both pathogenesis studies and vaccine design [13,14].

In the clinical field, the most convenient way to monitor both viral infection and vaccine response is by measuring antibodies in serum. ELISA is widely used in serological test due to its high sensitivity, reproducibility, and capacity for handling large numbers of serum samples. In recent years, efforts have been made to develop diagnostic tools for DAdV-3, including sandwich ELISA methods based on mAbs, indirect ELISA methods using recombinant Fiber2 proteins, and a competitive enzyme-linked immunosorbent assay (c-ELISA) [16,17,18]. Compared with indirect ELISA, both b-ELISA and c-ELISA offer comparable sensitivity but markedly higher specificity. All three formats are simple to run, easy to interpret, inexpensive and readily scalable. However, c-ELISA requires the preparation of enzyme-labeled antigens, which are intrinsically unstable and subject to batch-to-batch variation. In contrast, b-ELISA uses a stable monoclonal antibody–enzyme conjugate, thereby providing superior reproducibility and shelf-life. In pursuit of the highest specificity, long-term stability, and low cost, there remains a need for a highly specific and sensitive serological assay that can efficiently detect neutralizing antibodies against DAdV-3.

In this study, a b-ELISA for detection of neutralizing antibodies against DAdV-3 was developed, providing a valuable tool for clinical detection of DAdV-3 infections and for monitoring of antibody levels after vaccine immunization, which is crucial for effective disease prevention and control.

## 2. Materials and Methods

### 2.1. Viruses, Cells, Plasmids and Reagents

DAdV-3 SD2019 strain, isolated from sick Muscovy ducks in China in 2019, was maintained in our laboratory [19]. The LMH (CRL-2117) and SP2/0 myeloma (CRL-1581) cells were obtained from the American Type Culture Collection (ATCC). LMH cells were cultured in DMEM/F12 medium (Hyclone, Logan, UT, USA), supplemented with 10% fetal bovine serum (FBS) (Biowest, South America origin, Riverside, MO, USA), 100 U/mL of penicillin and 100 μg/mL of streptomycin (Sangon Biotech (Shanghai) Co., Ltd., Shanghai, China) at 37 °C with 5% CO_2_. The SP2/0 cells were cultured in DMEM, supplemented with 10% FBS, 100 U/mL of penicillin and 100 μg/mL of streptomycin at 37 °C with 5% CO_2_. Plasmids pCAGGS-Hexon-Flag, pCAGGS-Penton-Flag, pCAGGS-Fiber 1-Flag, and pCAGGS-Fiber 2-Flag were constructed, each containing the respective genes of Hexon, Penton, Fiber 1, and Fiber 2. ExFect Transfection Reagent was purchased from Nanjing Vazyme Biotech Co., Ltd. (Nanjing, China). The HRP-labeled, and FITC-labeled goat anti-mouse IgG antibodies (Jackson, West Grove, PA, USA), were purchased from Shanghai Nonin Biological Technology Co., Ltd. (Shanghai, China). The 4-week-old specific pathogen-free (SPF) female BALB/c mice were purchased from SPF Biotechnology Co., Ltd. (Suzhou, China). The hypoxanthine–aminopterin–thymidine (HAT) and hypoxanthine–thymidine (HT), were both purchased from Merck Limited, Shanghai. Specific sera against H9N2 avian influenza virus (H9N2 AIV), duck enteritis virus (DEV), fowl Adenovirus type 4, 8a, 8b and 11 (FAdV-4, FAdV-8a, FAdV-8b, FAdV-11), duck hepatitis A virus type 1 and 3 (DHAV-1, DHAV-3), duck Tembusu virus (DTMUV), novel duck reovirus (NDRV), duck Egg-Reducing Syndrome Virus (DERSV) and duck-origin goose parvovirus (D-GPV) were prepared and maintained in our laboratory, respectively.

### 2.2. Virus Concentration

The DAdV-3 SD2019 was propagated in LMH cells, cultured in T150 cm^2^ flasks, at a multiplicity of infection (MOI) of 0.01. The cells and culture were collected at 96 h post-infection, at which point 80% infected cells showed apparent cytopathic effects (CPEs). The mixtures were frozen–thawed three times, and the cell debris were removed by centrifugation at 3000× *g* for 10 min at 4 °C. 230 mL of supernatant was further ultracentrifugated at 150,000× *g* for 8 h and the ultracentrifugation pellets were gently redissolved in 1 mL of sterile PBS. The concentrated viruses, after identification, served dual purposes: as immunization antigens for antibody production and as coating antigens for ELISA.

### 2.3. Generation of mAbs Against DAdV-3

The mAbs against DAdV-3 were generated as described previously [20,21]. In brief, five 4-week-old female SPF BALB/c mice were inoculated subcutaneously with 50 mg of purified DAdV-3 mixed with complete Freund’s adjuvant (Sigma-Aldrich, St. Louis, MO, USA) for the first immunization. Then, the mice were boosted with the same dose of concentrated DAdV-3 mixed with Freund’s incomplete adjuvant (Sigma-Aldrich, St. Louis, MO, USA) every two weeks. The polyclonal antibody in sera of the immunized mice against the DAdV-3 were tested by the indirect ELISA every two weeks post second immunization. For the indirect ELISA, 96-well flat-bottom polystyrene microtiter plates were coated with inactivated and purified DAdV-3. When the serum of immunized mice was diluted 1000-fold and used as the primary antibody for indirect ELISA detection, the OD ratio of the immunized mouse serum to that of the blank mouse serum exceeded 2.0. Concurrently, the serum IFA titer reached 1:1000. At this juncture, the mouse was given an intraperitoneally injection of the same dose of concentrated DAdV-3 without adjuvant. Three days later, the fresh spleen cells of the mouse were isolated and merged with SP2/0 cells with polyethylene glycol solution (Sigma-Aldrich, St. Louis, MO, USA). The fused cells were plated in 96-well plates and maintained in DMEM, supplemented with 10% FBS and 1× Hybri-Max HAT Media Supplement (Sigma-Aldrich, St. Louis, MO, USA). The hybridoma cells secreting antibodies specific to DAdV-3 were initially detected by indirect ELISA. Selected hybridoma cell culture with high-titer was further confirmed by IFA using LMH cells infected with DAdV-3. The positive hybridoma cells were subcloned three times by limiting dilution method.

### 2.4. Indirect ELISA

An indirect ELISA was established using purified DAdV-3 to evaluate DAdV-3-specific antibodies in plasma. Briefly, concentrated DAdV-3 was serially diluted 1000-, 2000-, 4000-, 8000-, 16,000-, and 32,000-fold. Subsequently, 100 μL of each diluted DAdV-3 solution was used to coat 96-well ELISA plates, which then incubated overnight at 4 °C. The wells were blocked with 150 μL of 5% skim milk for 1 h at 37 °C. After discarding the blocking solution, the wells were incubated with 100 μL of diluted mouse sera (10-fold serial dilution in PBS) from immunized or naive mice for 1 h at 37 °C. The plates were washed three times with PBS containing 0.05% Tween-20 (PBST), and 100 μL of HRP-labeled goat anti-mouse IgG (1:4000 dilution) was added to each well, followed by incubation for 1 h at 37 °C. After washing three times with PBST, 100 μL of 3, 3′, 5, 5′-tetramethyl benzidine (TMB, Surmodics IVD, Inc., Eden Prairie, MN, USA) was added to each well and incubated at room temperature (RT) for 10 min. The reaction was stopped by adding 50 μL of 0.1 N sulfuric acid, and the absorbance was measured at 450 nm using a Varioskan Lux multimode microplate reader (Agilent-BioTek, Idealgo, Shanghai, China). The positive-to-negative (P/N) was calculated according to the formula: P/N = OD_450nm_ (sample)/OD_450nm_ (negative control). The antigen concentration that yields a P/N ratio greater than 2 at the highest dilution was selected as the optimal coating concentration. In this case, the optimal coating concentration of the antigen was 1:8000, and the optimal dilution ratio of the immune mouse serum was 1:400. The hybridoma cell culture supernatants were tested using the ELISA plate coated with the selected coating concentration.

### 2.5. IFA

The LMH cells, cultured on modular 96-well cell culture plates, were infected with DAdV-3 SD2019 or transfected with plasmids for IFA test. Briefly, the LMH cells were seeded onto the plates, 1 × 10^4^ cells per well, and the plates were cultured at 37 °C with 5% CO_2_. For virus-infected groups, the cells were inoculated with DAdV-3 SD2019 at a MOI of 0.01 on the following day. Meanwhile, the uninfected LMH cells were used as negative controls. For plasmids-transfected groups, 2 μg of plasmids were transfected into cells per well. At 72 h post-infection (hpi) or at 48 h post-transfection, the culture medium was carefully aspirated, cells were immobilized with 4% paraformaldehyde for 20 min at RT, washed three times in PBS (3 min per wash), the cells were permeabilized with 0.5% (*v*/*v*) Triton X-100 (Sigma-Aldrich, St. Louis, MO, USA) in PBS for 20 min at RT and blocked with 10% (*w*/*v*) bovine serum albumin (BSA; Sigma-Aldrich, St. Louis, MO, USA) in PBS for 20 min at RT. Then, the selected hybridoma cell supernatant was added to the wells and incubated for 1 h at 37 °C. After washing the wells three times with PBST, 100 μL of FITC-labeled goat anti-mouse IgG (diluted 1:400) was added and incubated for 1 h at 37 °C. After washing with PBST four times and covering with 50 μL of PBS, the cells were observed under an inverted fluorescence microscope (Carl Zeiss, Oberkochen, Germany).

### 2.6. Western Blotting (WB) Assay

The LMH cells were infected with DAdV-3 for 72 h and lysed with RIPA Lysis Buffer (Nonin Bio, Shanghai, China), supplemented with phenylmethanesulfonyl fluoridecontaining (PMSF), for 30 min on the ice. The lysates were then mixed with 1× SDS-PAGE Sample Loading Buffer (Beyotime Biotech Inc, Shanghai, China) and denatured at 99 °C for 10 min. The mixture was centrifuged for 1 min at 13,000× *g* at 4 °C to remove cell debris. The proteins in supernatant were separated under denaturing conditions in 10% SDS-polyacrylamide gel electrophoresis (SDS-PAGE, YamayBio LLC, Shanghai, China) and wet-transferred onto a nitrocellulose membrane (Liaoda, Shanghai Advantage Biological Co., Ltd., Shanghai, China). Meanwhile, the lysates were gently mixed with 1× non-denaturing and non-reducing protein loading buffer (WSHT Biotechnology Inc., Shanghai, China) by pipetting and kept on ice. The proteins in supernatant were separated under non-denaturing conditions using 10% non-denaturing PAGE (Solarbio, Beijing, China) and wet-transferred onto a nitrocellulose membrane. After blocking for 1 h at RT with 5% (*w*/*v*) skim milk in PBST, the membranes were probed with the appropriate primary antibody overnight at 4 °C under gentle shaking. After three washes with PBST, 5 min each time, the membranes were probed with HRP-conjugated goat anti-mouse IgG (1:2000 dilution) for 1 h at RT. Following four times of additional PBST washes, 5 min each time, protein bands were visualized using enhanced chemiluminescence (ECL) reagent (Share-Bio, Shanghai, China) and imaged with Tanon 5200 Multi Chemiluminescent Imager System (Tanon, Shanghai, China).

### 2.7. Preparation of Positive and Negative Sera Against DAdV-3

The DAdV-3 was inactivated by mixing with formaldehyde (at a final concentration of 0.2%) at 37 °C for 24 h. The inactivated virus was mixed with Montanide ISA 78VG adjuvant (Seppic, Croissy-Beaubourg, France) at a volume ratio of 2.6:7.4 and was emulsified to prepare an inactivated vaccine. Four 5-day-old SPF ducks were initially immunized subcutaneous (s.c.) with 1 mL of inactivated vaccine. The ducks were subsequently given an intramuscularly (i.m.) booster immunization with the inactivated vaccine at 21 days post initial immunization. Duck sera were collected two weeks after the second immunization. The titers of sera were determined using the SNT, with titers more than 1:2 considered positive for anti-DAdV-3 antibodies. Sera from three 5-week-old SPF ducks, raised in isolators, were collected and used as negative controls, with SNT titers less than 1:2.

### 2.8. SNT

The sera (deactivated at 56 °C for 30 min) or mAb samples were subjected to a series of 2-fold dilutions by mixing 500 μL of each with 500 μL of sterile PBS. Combined 500 μL of every dilution with 500 μL of DAdV-3 SD2019 (100 TCID_50_) at a volume ratio of 1:1 and incubate at 37 °C for 1 h. An aliquot (200 μL) of each mixture was layered onto confluent LMH cell monolayers in a 96-well plate and allowed to adsorb for 2 h at 37 °C. After washing three times with sterile PBS, the cells were maintained in DMEM/F12 containing 2% FBS at 37 °C with 5% CO_2_ for 6 days. Neutralization titers of sera and mAbs were then calculated by the Reed–Muench method [20].

### 2.9. Development of the Blocking ELISA

The optimal dilutions of coating DAdV-3 antigen and the mAb were established via checkerboard titration. ELISA plates were coated overnight at 4 °C with purified DAdV-3 antigen serially diluted 1000-, 2000-, 4000-, 8000-, 16,000-, and 32,000-fold in 0.1 M carbonate–bicarbonate buffer (pH 9.6). The plates were then washed three times with PBST and blocked with 100 μL of 5% skim milk for 1.5 h at 37 °C. Duck serum samples (1:10 dilution with PBS) were added to the wells and incubated for 1 h at 37 °C. After washing three times with PBST, 100 μL of mAb (diluted 100- to 51,200-fold with the sample diluent) was added and incubated for 1 h at 37 °C. Following three more washes with PBST, HRP-labeled goat anti-mouse IgG (1:4000 dilution with PBS) was added and incubated for 1 h at 37 °C. After washing three times with PBST, 100 μL of TMB was added, and the plates were incubated in the dark at RT for 10 min. The reaction was stopped by adding 50 μL of 0.1 N sulfuric acid. The optical density (OD) was measured at 450 nm, and the percent inhibition (PI) value was calculated using the formula: PI (%) = [1 − (OD_450nm_ of test sample/OD_450nm_ of negative control serum)] × 100%. Each plate contained duplicate positive and negative controls. The optimal coating concentration of the antigen for the b-ELISA was determined to be 1:1000, and the optimal dilution ratio of the mAb was 1:1600.

### 2.10. Determination of the Cut-Off Value

To determine the cut-off value for differentiating between positive and negative serum samples, eighty-four duck serum samples from DAdV-3-negative duck farms were analyzed. The cut-off criteria were defined as follows: Samples with a PI value ≤ $\bar{X}$ + 2SD were classified as negative, while those with a PI ≥ $\bar{X}$ + 3SD were considered positive. Samples with PI values falling between these two thresholds were deemed suspicious and required retesting. Upon retesting, samples with a PI < $\bar{X}$ + 3SD were classified as negative. This approach ensured that the majority of truly negative samples were accurately categorized, thereby minimizing the false-positive rate while maintaining high specificity without sacrificing sensitivity.

### 2.11. Specificity Test

To assess the specificity of the b-ELISA, thirteen types of positive serum samples were tested. These samples were reactive against DAdV-3, H9N2 AIV, DEV, FAdV-4, FAdV-8a, FAdV-8b, FAdV-11, DHAV-1, DHAV-3, DTMUV, NDRV, DERSV, and D-GPV, respectively.

### 2.12. Sensitivity Test

To evaluate the sensitivity of the b-ELISA for DAdV-3, positive standard serum samples were subjected 2-fold serially dilution, while negative standard serum samples were diluted at a ratio of 1:2. Both the b-ELISA and the SNT were performed concurrently on these samples with varying dilutions, and the PI values and neutralization titers were determined. The highest dilution factors that still produced positive results were compared between the b-ELISA and the SNT.

### 2.13. Field Sample Application

To assess the field applicability of the b-ELISA, 90 field serum samples collected from six different duck farms were tested using the b-ELISA.

## 3. Results

### 3.1. Generation and Characterization of DAdV-3-Specific mAbs

**Figure 2:**
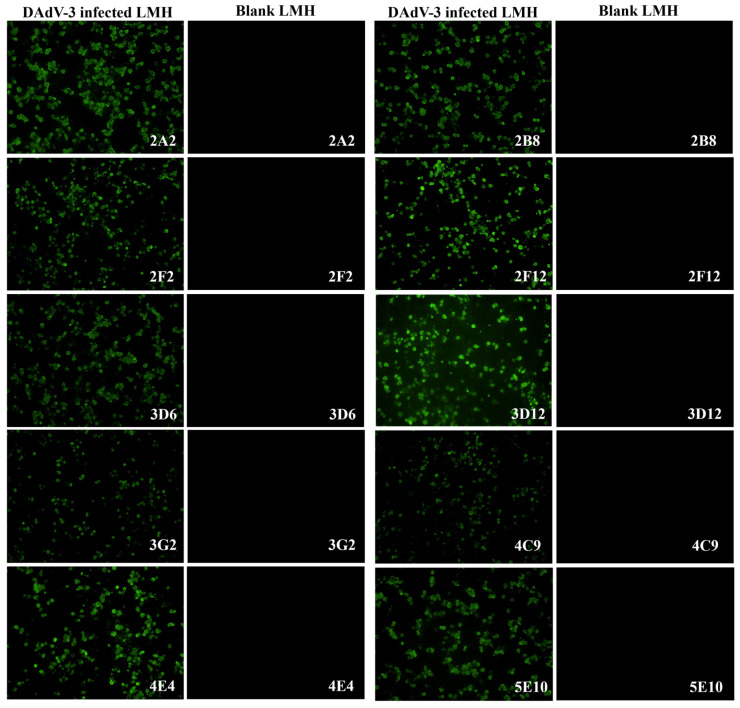
Characterization of ten monoclonal antibodies by IFA. To characterize the ten mAbs against DAdV-3, an IFA was performed using LMH cells infected with DAdV-3. The cell supernatants of the ten mAbs were used as the primary antibodies to incubate both the positive cell plates of LMH infected with DAdV-3 and the blank LMH cell plates. Under fluorescence microscopy, all ten mAbs specifically reacted with the infected cells in the positive cell plates emitting green fluorescence, while no reaction was observed with the blank LMH cells.

To generate DAdV-3-specific mAbs, five 4-week-old BALB/c mice were immunized with concentrated DAdV-3 SD2019. Following cell fusion, the supernatants of hybridoma cells were screened using indirect ELISA with purified DAdV-3 as the coating antigen and IFA on DAdV-3-infected LMH cells (Figure 2). Ten monoclonal hybridoma cell lines producing DAdV-3-specific antibodies were successfully identified and purified with three rounds of subcloning.

To assess whether anti-DAdV-3 neutralizing sera could block the epitopes targeted by these mAbs, a blocking ELISA was developed and performed. The results revealed that four mAbs (2F12, 3D6, 3D12, and 5E10) exhibited PI values exceeding 50% (Table 1), confirming their neutralizing activity. Among them, the PI value of the 2F12 strain was the highest, reaching 75.21%. So, the 2F12 cell line was subsequently used to produce ascites in mice, yielding a neutralizing titer of 1:794.

**Figure 3:**
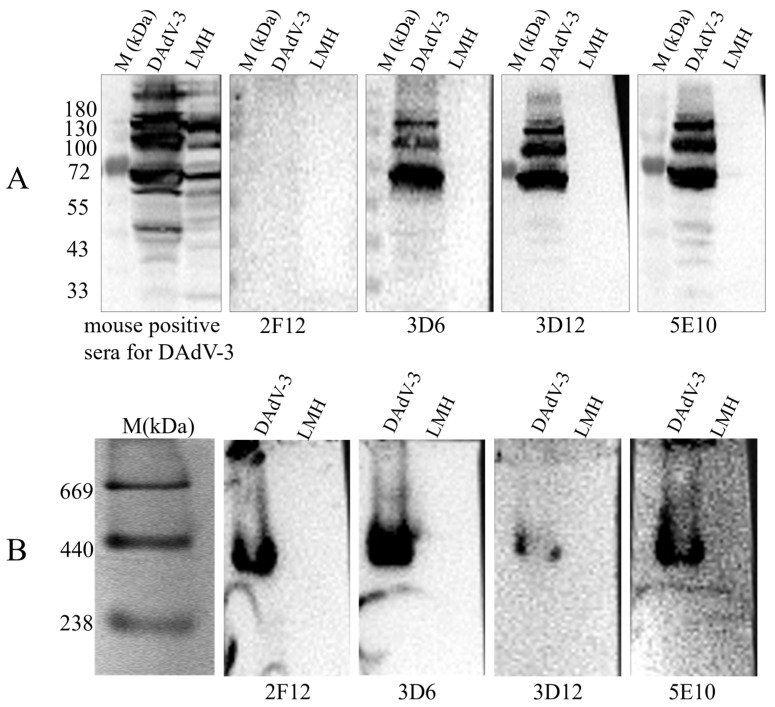
Detection of the reactivity of mAb 2F12, 3D6, 3D12 and 5E10 with DAdV-3 viral proteins by Western blotting. (**A**) Denaturing WB and (**B**) non-denaturing WB analysis of mAb 2F12, 3D6, 3D12 and 5E10.

To determine the WB reactivity of the mAbs 2F12, 3D6, 3D12, and 5E10, LMH cells infected with DAdV-3 were lysed and split into denatured and native protein samples. Both no-reduced WB (without SDS/reducing agent) and standard SDS-PAGE/WB were run simultaneously. We found that, under the denaturing WB condition, 2F12 group did not produce specific bands, while 5E10 and 3D12 groups did, with multiple bands likely due to glycosylation or other modifications (Figure 3). In contrast, non-denaturing WB revealed specific bands of consistent size (238–440 kDa) for all four mAbs (Figure 3). The results indicated that mAb 5E10 and 3D12 target linear epitopes, whereas mAb 2F12 targets a conformational epitope. Additionally, the band size is consistent with the molecular weitht of trimeric Hexon (~327 kDa), indicating that the 2F12 only binds to the native trimeric structure of the Hexon of DAdV-3.

**Figure 4:**
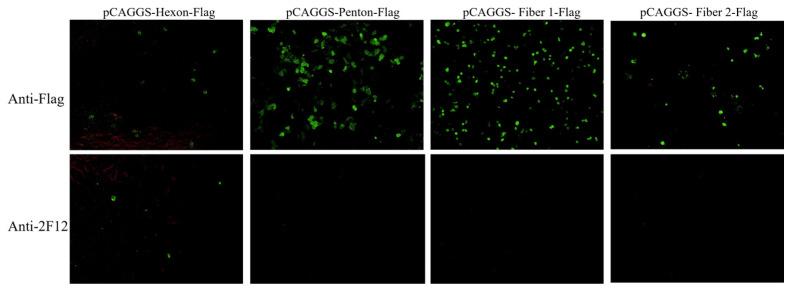
Determine the interaction results between the monoclonal antibody 2F12 and the surface protein through IFA.

To identify which surface protein of DAdV-3 interacts with 2F12, plasmids pCAGGS-Hexon-Flag, and pCAGGS-Penton-Flag, pCAGGS-Fiber 1-Flag, pCAGGS-Fiber 2-Flag, were constructed and transfected into LMH cells. IFA was performed using anti-Flag antibody and 2F12 as primary antibodies. The results showed that all groups treated with anti-Flag antibody exhibited specific green fluorescence (Figure 4). However, in the groups treated with 2F12, only the Hexon-expressing cells showed specific green fluorescence, despite the relatively low expression level of Hexon. The other three groups did not show specific green fluorescence (Figure 4), indicating that 2F12 specifically targets Hexon.

### 3.2. Establishment of a Blocking ELISA for DAdV-3 Antibody Detection

A blocking ELISA was established that exploited the capacity of anti-DAdV-3 serum to compete with and block the neutralizing epitope recognized by mAb 2F12. To determine the assay’s cut-off values, eighty-four duck serum samples, collected from four DAdV-3-free farms in Jiangsu, Zhejiang, Shandong and Anhui, were tested. The mean PI ($\bar{X}$) of these negative sera was 7.14%, and the SD was 4.83%. Based on these thresholds, the cut-off values were determined as follows: PI ≥ 21.62% ($\bar{X}$ + 3SD), the serum was considered positive, PI ≤ 16.79% ($\bar{X}$ + 2SD), the serum was considered negative, 16.79% < PI < 21.62%, the serum should be tested again. If the repeat PI value remained <21.62%, the sample was classified as negative (Table 2).

### 3.3. Specificity and Sensitivity of the B-ELISA

**Figure 5:**
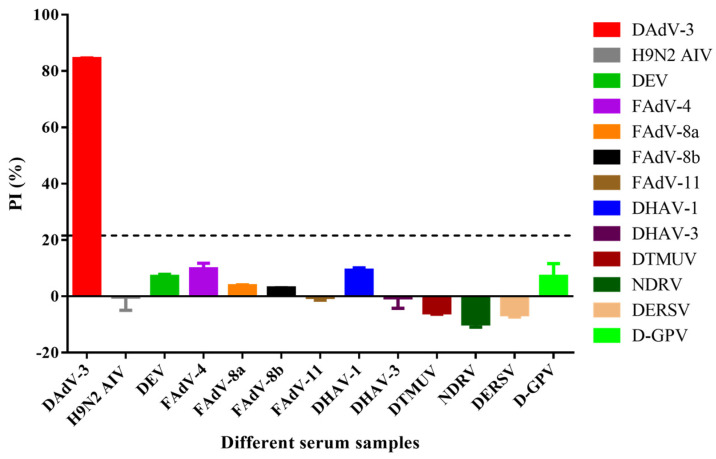
Specificity of the b-ELISA to anti-DAdV-3 serum. Thirteen sera against different viruses were investigated. The PI value of anti-DAdV-3 serum reached a maximum of 84.44%, while the other antisera against H9N2 AIV, DEV, FAdV-4, FAdV-8a, FAdV-8b, FAdV-11, DHAV-1, DHAV-3, DTMUV, NDRV, DERSV and D-GPV ranged from −9.77% to 9.57%, were all negative. The dotted line referred to the cut off value of 21.62%.

To evaluate the specificity of the b-ELISA, sera against several common duck pathogens and fowl adenoviruses, including DAdV-3, H9N2 AIV, DEV, FAdV-4, FAdV-8a, FAdV-8b, FAdV-11, DHAV-1, DHAV-3, DTMUV, NDRV, DERSV, and D-GPV were tested, respectively. The results demonstrated high specificity for DAdV-3 antibody, with the anti-DAdV-3 serum yielding a maximal PI value of 84.44%, while sera against other viruses showed PI values below 16.79% (Figure 5). These findings confirmed that the b-ELISA specifically detects anti-DAdV-3 antibodies without significant cross-reactivity against sera from other duck pathogens and fowl adenoviruses.

Additionally, the sensitivity of the b-ELISA was compared to SNT using serial dilutions of anti-DAdV-3 serum. The b-ELISA detected antibodies at a 16-fold higher dilution than SNT (Table 3), demonstrating improved sensitivity. The results indicated that the b-ELISA is more sensitive in detecting DAdV-3 antibodies compared to the traditional SNT.

### 3.4. Field Application of the b-ELISA

To evaluate the b-ELISA for field applicability, 90 duck sera obtained from six duck farms were analyzed. The test results showed a clear contrast between the infected farms (A and C), and the uninfected farms (B, D, E and F). Serum samples from A farm showed 100% (15/15) positive (Table 4), indicating a complete infection. Out of 15 serum samples tested, only 6 were positive (Table 4), resulting in a 40% positive, suggesting C farm was in the midst of an infection period. Samples from B, D, E and F were all negative (Table 4), which means ducks in these farms were free of the DAdV-3 infection.

## 4. Discussion

Avian adenoviruses are globally widespread pathogens that pose significant threats to poultry industry [1,6,21,22]. They not only directly impact the health and productivity of poultry but also raise concerns regarding food safety and public health [23]. DAdV-3 was recently identified as a type of duck adenovirus that has rapidly became a major concern in China. The development of a specific serological assay for DAdV-3 infections and monitoring post-vaccination antibody levels is therefore of critical importance for effective disease prevention and control.

As the main component of the capsid, Hexon possesses specific antigenic determinants and is crucial for inducing neutralizing antibodies [12]. In this study, we successfully generated ten hybridoma cell lines producing mAbs specific to DAdV-3, namely 2A2, 2F2, 2B8, 2F12, 3D6, 3D12, 3G2, 4E4, 4C9, and 5E10. Among these, four mAbs (2F12, 3D6, 3D12, and 5E10) demonstrated a blocking rate of over 50% in the b-ELISA, indicating their neutralizing potential. The 2F12 strain, which exhibited the highest blocking rate, was selected for further development of the b-ELISA. The neutralizing potency of the ascites derived from the 2F12 cell line reached up to 1:794, indicating its neutralizing efficacy against DAdV-3. To assess the WB reactivity of the 2F12 against DAdV-3, the band ranged from 238 to 440 kDa only appeared under no-reduced conditions. This band size was consistent with the molecular weight of trimeric Hexon (~327 kDa), indicating that the 2F12 binds to the native structure of the Hexon of DAdV-3. Among the four major surface proteins (Hexon, Penton, Fiber 1, and Fiber 2) of DAdV-3 expressed in LMH cells, only the Hexon group showed specific green fluorescence. Despite its large molecular weight, which led to low expression levels in LMH cells, Hexon still showed specific fluorescence with 2F12, confirming their interaction.

Several serological methods have been established for the detection of antibodies against DAdV-3 [16,18]. The indirect ELISA relies on the binding of all serum antibodies directed against the full range of viral proteins, offering higher sensitivity, yet it remains unclear whether the antibodies detected possess neutralizing activity. The competitive ELISA (c-ELISA), based on Fiber 2 protein and an HRP-labeled mAb, provides a useful tool for large-scale serological surveillance of DAdV-3 in ducks. However, this method requires labeling of the prepared mAb, which demands high technical proficiency. Moreover, although this method detects antibodies against Fiber 2, it has not been validated whether these antibodies possess neutralizing activity. In our study, the b-ELISA we developed using a neutralizing mAb 2F12 to capture serum antibodies, establishing a direct correlation with challenge protection. This approach is particularly advantageous for evaluating vaccine efficacy.

The sensitivity and specificity of the established b-ELISA were assessed. For the specificity panel, we selected several duck-origin viruses and fowl adenoviruses. In China. DAdV-3 is the dominant adenovirus in duck flocks, while recent reports have documented FAdV-4 infections in ducks [24]. Other fowl adenoviruses, although primarily chicken pathogens, were also included. The assay demonstrated exclusive specificity for DAdV-3, with no cross-reactivity against other duck and fowl adenoviruses such as H9N2 AIV, DEV, FAdV-4, FAdV-8a, FAdV-8b, FAdV-11, DHAV-1, DHAV-3, DTMUV, NDRV, DERSV, and D-GPV. This high specificity is crucial for accurate diagnosis and epidemiological surveillance. Moreover, the b-ELISA exhibited superior sensitivity compared to the SNT, detecting antibodies at a 16-fold higher dilution than the SNT. The enhanced sensitivity allows for more efficient detection of DAdV-3 antibodies, even at lower concentrations. The results highlight the potential of the b-ELISA as a robust, specific, and sensitive tool for DAdV-3 serological surveillance and diagnosis.

The field application of the b-ELISA was further validated using 90 serum samples collected from six duck farms. The results showed a different seropositivity rate for DAdV-3 infection different duck farms, demonstrating that the b-ELISA established in this study is well-suitable for field detection.

In summary, a highly specific and sensitive b-ELISA was established for the detection of DAdV-3 neutralizing antibodies in this study. And the assay can be effectively applied in epidemiological investigation and vaccine immunization efficacy evaluation.

## Figures and Tables

**Table 1:** Characterization of ten monoclonal antibodies by b-ELISA.

Monoclonal Antibody ^a^	Positive Serum OD_450nm_ (P) ^b^	Negative Serum OD_450nm_ (N) ^c^	PI (%) ^d^
2A2	1.13	1.20	5.83
2B8	1.19	1.16	−2.59
2F2	1.95	1.86	−4.84
2F12	0.59	2.38	75.21
3D6	1.57	3.38	53.55
3D12	0.58	2.06	71.84
3G2	2.97	3.49	14.90
4E4	3.14	3.36	6.55
4C9	1.19	1.08	−10.19
5E10	0.80	2.17	63.13

Note: ^a^: The blocking ELISA was used to characterize ten mAbs. The cell supernatants of mAbs were used as the second antibodies to incubate the ELISA plates, which had been previously coated with DAdV-3 antigen and incubated with positive and negative duck sera. ^b^: (P) The OD_450nm_ was measured for positive sera. Diluted the positive serum at a ratio of 1:10. ^c^: (N) The OD_450nm_ was measured for negative sera. Diluted the negative serum at a ratio of 1:10. ^d^: (PI) The PI was calculated using the formula: PI (%) = (1 − (P/N)) × 100%.

**Table 2:** Cut-off value of the b-ELISA antibody detection method for DAdV-3.

PI Values from Eighty-Four Clinical Negative Duck Sera								
PI (%)	3.67	5.36	4.25	8.02	5.06	8.67	15.58	12.60
	2.01	6.69	9.51	6.49	6.20	16.36	5.62	2.56
	15.23	1.33	6.46	9.42	5.52	5.71	8.70	3.28
	15.42	6.56	10.23	0.58	−0.29	12.76	7.08	2.63
	6.98	5.13	−0.10	17.95	3.05	5.16	7.24	−0.75
	−4.32	2.18	3.15	8.21	11.66	5.97	4.58	9.16
	20.45	−2.47	2.95	−0.52	4.16	6.04	14.25	12.37
	10.16	11.82	3.02	5.16	7.24	6.17	9.68	17.14
	10.91	12.82	3.73	8.70	9.12	9.22	4.48	3.02
	8.38	10.23	3.90	9.81	11.72	4.68	7.66	16.14
	7.31	7.50	3.15	5.13				
$\bar{X}$ (%)	7.14							
SD (%)	4.83							

Note: PI ≥ $\bar{X}$ + 3SD, the serum is considered positive; PI ≤ $\bar{X}$ + 2SD, the serum is considered negative; $\bar{X}$ + 2SD < PI < $\bar{X}$ + 3SD, the serum should be tested again. If the retesting PI value remains < $\bar{X}$ + 3SD, the sample is classified as negative.

**Table 3:** Sensitivity comparison between the b-ELISA and SNT.

Serum Dilution ^a^	PI (%)	SNT
2^6^	84.02 (+) ^b^	++ ^d^
2^7^	78.30 (+)	++
2^8^	72.41 (+)	++
2^9^	62.48 (+)	++
2^10^	44.67 (+)	+− ^e^
2^11^	39.93 (+)	−− ^f^
2^12^	30.64 (+)	−−
2^13^	22.64 (+)	−−
2^14^	13.35 (−) ^c^	−−
2^15^	6.89 (−)	−−
2^16^	3.67 (−)	−−

Note: ^a^, Dilution in PBS. ^b^, (+) Positive result. ^c^, (−) Negative result. ^d^, (++) Complete neutralization. ^e^, (+−) Partial neutralization. ^f^, (−−) No neutralization.

**Table 4:** Field serum samples test.

Duck Farm	Positive Results	Negative Results	Total Number	Positive Detection Rate
A	15	0	15	100%
B	0	15	15	0%
C	6	9	15	40%
D	0	15	15	0%
E	0	15	15	0%
F	0	15	15	0%

Note: Diluted the negative serum at a ratio of 1:10.

## Data Availability

The original contributions presented in this study are included in the article/Supplementary Material. Further inquiries can be directed to the corresponding authors.

## Supplementary Materials

The following supporting information can be downloaded at: https://www.mdpi.com/article/10.3390/microorganisms13112607/s1. Table S1: Cut-off value of blocking ELISA antibody detection method for DAdV-3; Table S2: Sensitivity comparison between the blocking ELISA and SNT; Table S3: Field serum samples test; Table S4: Specificity of the b-ELISA to anti-DAdV-3 serum.
