# Supplementary material for: Development of a Blocking ELISA for Detection of Serum Neutralizing Antibodies Against Duck Adenovirus Type 3
Source: Microorganisms. 2025 Nov 16;13(11):2607. doi: 10.3390/microorganisms13112607 (PMC12654197; doi:10.3390/microorganisms13112607)
Supplement: Supplementary file 1 [file microorganisms-13-02607-s001.zip › Table S1.pdf]

Table S1: Cut-off value of blocking ELISA antibody detection method for DAdV-3

	$OD_{450\ nm}$ from eighty-four clinical negative duck sera							
$OD_{450\ nm}$	2.00	1.97	1.99	1.91	1.97	1.90	1.76	1.82
	2.04	1.94	1.88	1.94	1.95	1.74	1.96	2.03
	1.76	2.05	1.95	1.88	1.97	1.96	1.90	2.01
	1.76	1.94	1.87	2.07	2.09	1.81	1.93	2.03
	1.93	1.97	2.08	1.71	2.02	1.97	1.93	2.10
	2.17	2.03	2.01	1.91	1.84	1.96	1.98	1.89
	1.65	2.13	2.02	2.09	1.99	1.95	1.78	1.82
	1.87	1.83	2.02	1.97	1.93	1.95	1.88	1.72
	1.85	1.81	2.00	1.90	1.89	1.89	1.99	2.02
	1.91	1.87	2.00	1.88	1.84	1.98	1.92	1.74
	1.93	1.92	2.01	1.97				
Negative control	2.04	2.12						
Positive control	0.41	0.39						
