# Supplementary material for: Development of a Blocking ELISA for Detection of Serum Neutralizing Antibodies Against Duck Adenovirus Type 3
Source: Microorganisms. 2025 Nov 16;13(11):2607. doi: 10.3390/microorganisms13112607 (PMC12654197; doi:10.3390/microorganisms13112607)
Supplement: Supplementary file 1 [file microorganisms-13-02607-s001.zip › Table S2.pdf]

Table S2: Sensitivity comparison between the blocking ELISA and SNT

Serum dilution [a]	Positive serum $OD_{450\ nm}$ (P) [g]		Negative serum $OD_{450\ nm}$ (N) [h]		PI (%)	SNT
2^{6}	0.253	0.249	1.56	1.58	84.02 (+) [b]	++ [d]
2^{7}	0.341	0.340	1.56	1.58	78.30 (+)	++
2^{8}	0.437	0.429	1.56	1.58	72.41 (+)	++
2^{9}	0.593	0.585	1.56	1.58	62.48 (+)	++
2^{10}	0.868	0.869	1.56	1.58	44.67 (+)	+- [e]
2^{11}	0.947	0.939	1.56	1.58	39.93 (+)	-- [f]
2^{12}	1.097	1.081	1.56	1.58	30.64 (+)	--
2^{13}	1.149	1.280	1.56	1.58	22.64 (+)	--
2^{14}	1.422	1.299	1.56	1.58	13.35 (-) [c]	--
2^{15}	1.466	1.458	1.56	1.58	6.89 (-)	--
2^{16}	1.515	1.510	1.56	1.58	3.67 (-)	--
