# Supplementary material for: Development of a Blocking ELISA for Detection of Serum Neutralizing Antibodies Against Duck Adenovirus Type 3
Source: Microorganisms. 2025 Nov 16;13(11):2607. doi: 10.3390/microorganisms13112607 (PMC12654197; doi:10.3390/microorganisms13112607)
Supplement: Supplementary file 1 [file microorganisms-13-02607-s001.zip › Table S3.pdf]

Table S3: Field serum samples test

samples	PI in b-ELISA	$OD_{450\ nm}$	samples	PI in b-ELISA	$OD_{450\ nm}$	samples	PI in b-ELISA	$OD_{450\ nm}$	Negative control $OD_{450\ nm}$	Positive control $OD_{450\ nm}$
A-1	83.59%	0.23	C-1	51.26%	0.69	E-1	16.59%	1.18	1.40	0.24
A-2	62.88%	0.53	C-2	42.31%	0.82	E-2	12.22%	1.24	1.44	0.25
A-3	68.85%	0.44	C-3	21.81%	1.11	E-3	7.10%	1.32		
A-4	73.93%	0.37	C-4	5.58%	1.34	E-4	16.27%	1.19		
A-5	58.19%	0.59	C-5	0.07%	1.42	E-5	11.65%	1.25		
A-6	75.17%	0.35	C-6	6.64%	1.32	E-6	5.79%	1.33		
A-7	80.70%	0.27	C-7	6.50%	1.32	E-7	0.50%	1.41		
A-8	77.26%	0.32	C-8	13.18%	1.23	E-8	13.93%	1.22		
A-9	46.29%	0.76	C-9	8.03%	1.30	E-9	9.70%	1.28		
A-10	60.16%	0.56	C-10	11.15%	1.26	E-10	5.75%	1.34		
A-11	68.53%	0.45	C-11	14.28%	1.21	E-11	1.81%	1.39		
A-12	57.53%	0.60	C-12	16.13%	1.19	E-12	2.45%	1.38		
A-13	81.45%	0.26	C-13	27.26%	1.03	E-13	8.31%	1.30		
A-14	55.75%	0.63	C-14	46.29%	0.76	E-14	4.58%	1.35		
A-15	67.85%	0.46	C-15	30.16%	0.99	E-15	6.25%	1.33		
B-1	12.54%	1.24	D-1	3.09%	1.37	F-1	2.95%	1.38		
B-2	12.75%	1.24	D-2	10.23%	1.27	F-2	0.00%	1.42		
B-3	9.70%	1.28	D-3	-2.52%	1.45	F-3	4.90%	1.35		
B-4	2.06%	1.39	D-4	15.49%	1.20	F-4	3.55%	1.37		
B-5	8.70%	1.29	D-5	16.27%	1.19	F-5	0.25%	1.41		
B-6	13.43%	1.23	D-6	13.68%	1.22	F-6	11.40%	1.26		
B-7	12.97%	1.23	D-7	9.20%	1.29	F-7	1.88%	1.39		
B-8	10.52%	1.27	D-8	10.27%	1.27	F-8	1.10%	1.40		
B-9	5.65%	1.34	D-9	7.85%	1.31	F-9	3.66%	1.37		
B-10	10.05%	1.27	D-10	9.41%	1.28	F-10	-3.73%	1.47		
B-11	12.90%	1.23	D-11	14.46%	1.21	F-11	-2.34%	1.45		
B-12	10.73%	1.26	D-12	20.11%	1.13	F-12	-6.29%	1.51		
B-13	7.53%	1.31	D-13	11.23%	1.26	F-13	-3.37%	1.46		
B-14	8.67%	1.29	D-14	8.06%	1.30	F-14	12.90%	1.23		
B-15	7.85%	1.31	D-15	11.51%	1.25	F-15	8.67%	1.29		
