# Supplementary material for: Development of a Blocking ELISA for Detection of Serum Neutralizing Antibodies Against Duck Adenovirus Type 3
Source: Microorganisms. 2025 Nov 16;13(11):2607. doi: 10.3390/microorganisms13112607 (PMC12654197; doi:10.3390/microorganisms13112607)
Supplement: Supplementary file 1 [file microorganisms-13-02607-s001.zip › Table S4.pdf]

Table S4: Specificity of the b-ELISA to anti-DAdV-3 serum.

Pathogenic serum	Positive serum $OD_{450\ nm}$		PI in b-ELISA		Average PI value	Result determination
DEV	2.12	2.06	6.61%	7.62%	7.12%	-
AIV	2.2	2.31	3.08%	-3.59%	-0.25%	-
FAdV-4	2.08	1.98	8.37%	11.21%	9.79%	-
FAdV-8a	2.18	2.15	4.00%	3.51%	9.79%	-
FAdV-8b	2.20	2.16	2.98%	3.04%	9.79%	-
FAdV-11	2.26	2.25	0.34%	-1.05%	9.79%	-
DHAV-1	2.07	2.01	8.68%	9.87%	8.68%	-
DHAV-3	2.22	2.3	2.20%	-3.14%	-0.47%	-
DTMUV	2.41	2.35	-6.17%	-5.38%	-5.77%	-
NDRV	2.51	2.43	-10.57%	-8.97%	-9.77%	-
DERSV	2.43	2.36	-7.05%	-5.83%	-6.44%	-
D-GPV	2.18	2	3.96%	10.31%	7.14%	-
Negative control	2.27	2.23	0.00%	0.00%	0.00%	-
Positive control	0.35	0.35	84.58%	84.30%	84.44%	+
